# Functional Aplasia of the Contralateral A1 Segment Influences Clinical Outcome in Patients with Occlusion of the Distal Internal Carotid Artery

**DOI:** 10.3390/jcm11051293

**Published:** 2022-02-26

**Authors:** Sebastian Fischer, Lukas Goertz, Charlotte S. Weyland, Ali Khanafer, Christoph J. Maurer, Hanna Zimmermann, Thomas David Fischer, Hanna Styczen, Benjamin Tan, Maria Alexandrou, Donald Lobsien, Elmar Lobsien, Maximilian Thormann, Lukas Meyer, Nuran Abdullayev, Jens Fiehler, Anastasios Mpotsaris, Panagiotis Papanagiotou, Leonard Yeo, Cornelius Deuschl, Thomas Liebig, Ansgar Berlis, Hans Henkes, Markus Möhlenbruch, Volker Maus

**Affiliations:** 1 Institute of Diagnostic and Interventional Radiology, Neuroradiology and Nuclear Medicine, Knappschaftskrankenhaus Bochum, Ruhr-University Bochum, 44892 Bochum, Germany; sebastian.fischer@kk-bochum.de; 2 Department of Diagnostic and Interventional Radiology, University Hospital Cologne, 50937 Cologne, Germany; lukas.goertz@uk-koeln.de (L.G.); nuran.abdullayev@uk-koeln.de (N.A.); 3 Department of Diagnostic and Interventional Neuroradiology, University Hospital Heidelberg, 69120 Heidelberg, Germany; charlotte.weyland@med.uni-heidelberg.de (C.S.W.); markus.moehlenbruch@med.uni-heidelberg.de (M.M.); 4 Neuroradiological Clinic, Klinikum Stuttgart, 70174 Stuttgart, Germany; mr-khanafer@hotmail.com (A.K.); hhhenkes@aol.com (H.H.); 5 Department of Diagnostic and Interventional Neuroradiology, University Hospital Augsburg, 86156 Augsburg, Germany; christoph.maurer@uk-augsburg.de (C.J.M.); ansgar.berlis@uk-augsburg.de (A.B.); 6 Institute for Diagnostic and Interventional Neuroradiology, University Hospital München-Großhadern, 81377 Munich, Germany; hanna.zimmermann@med.uni-muenchen.de (H.Z.); thomas.fischer@med.uni-muenchen.de (T.D.F.); thomas.liebig@med.uni-muenchen.de (T.L.); 7 Institute for Diagnostic and Interventional Radiology and Neuroradiology, University Hospital Essen, 45147 Essen, Germany; hanna.styczen@uk-essen.de (H.S.); cornelius.deuschl@uk.essen.de (C.D.); 8 Division of Neurology, Department of Medicine, Yong Loo Lin School of Medicine, National University of Singapore, National University Health System, Singapore 119077, Singapore; benjaminyqtan@gmail.com (B.T.); leonardyeoll@gmail.com (L.Y.); 9 Department of Diagnostic and Interventional Neuroradiology, Klinikum Bremen-Mitte, 28211 Bremen, Germany; maria.alexandrou@klinikum-bremen-mitte.de (M.A.); papanagiotou@me.com (P.P.); 10 Department of Diagnostic and Interventional Radiology and Neuroradiology, Helios General Hospital Erfurt, 99089 Erfurt, Germany; donald.lobsien@helios-gesundheit.de; 11 Department of Neurology, Helios General Hospital Erfurt, 99089 Erfurt, Germany; elmar.lobsien@helios-gesundheit.de; 12 Department of Diagnostic and Interventional Radiology, University Hospital Magdeburg, 39120 Magdeburg, Germany; maximilian.thormann@med.ovgu.de (M.T.); anastasios.mpotsaris@med.ovgu.de (A.M.); 13 Department of Diagnostic and Interventional Neuroradiology, University Hospital Hamburg-Eppendorf, 20246 Hamburg, Germany; lu.meyer@uke.de (L.M.); fiehler@uke.de (J.F.); 14 Department of Radiology, Aretaieion University Hospital, National and Kapodistrian University of Athens, 10679 Athens, Greece

**Keywords:** mechanical thrombectomy, acute ischemic stroke, ICA occlusion

## Abstract

Background: The importance of an A1 aplasia remains unclear in stroke patients. In this work, we analyze the impact of an A1 aplasia contralateral to an acute occlusion of the distal internal carotid artery (ICA) on clinical outcomes. Methods: We conducted a retrospective study of consecutive stroke patients treated with mechanical thrombectomy at 12 tertiary care centers between January 2015 and February 2021 due to an occlusion of the distal ICA. Functional A1 aplasia was defined as the absence of A1 or hypoplastic A1 (>50% reduction to the contralateral site). Functional independence was measured by the modified Rankin Scale (mRS ≤ 2). Results: In total, 81 out of 1068 (8%) patients had functional A1 aplasia contralateral to distal ICA occlusion. Patients with functional contralateral A1 aplasia were more severely affected on admission (median NIHSS 18, IQR 15–23 vs. 17, IQR 13–21; aOR: 0.672, 95% CI: 0.448–1.007, *p* = 0.054) and post-interventional ischemic damage was larger (median ASPECTS 5, IQR 1–7, vs. 6, IQR 3–8; aOR: 1.817, 95% CI: 1.184–2.789, *p* = 0.006). Infarction occurred more often within the ipsilateral ACA territory (20/76, 26% vs. 110/961, 11%; aOR: 2.482, 95% CI: 1.389–4.437, *p* = 0.002) and both ACA territories (8/76, 11% vs. 5/961, 1%; aOR: 17.968, 95% CI: 4.979–64.847, *p* ≤ 0.001). Functional contralateral A1 aplasia was associated with a lower rate of functional independence at discharge (6/81, 8% vs. 194/965, 20%; aOR: 2.579, 95% CI: 1.086–6.122, *p* = 0.032) and after 90 days (5/55, 9% vs. 170/723, 24%; aOR: 2.664, 95% CI: 1.031–6.883, *p* = 0.043). Conclusions: A functional A1 aplasia contralateral to a distal ICA occlusion is associated with a poorer clinical outcome.

## 1. Introduction

Mechanical thrombectomy (MT) is the standard treatment for patients suffering from acute ischemic stroke (AIS) due to intracranial large vessel occlusion (LVO) in the anterior circulation based on several randomized controlled trials in 2015 [1]. Nearly 20% of the affected individuals presented with an occlusion of the distal internal carotid artery (ICA), including all different conformations of “carotid-I”, “carotid-L” and “carotid-T”, or involving the M1 segment of the middle cerebral artery (MCA) [2]. Such distal ICA occlusions are often accompanied by a poor clinical outcome and high mortality, which might be traced to several factors, e.g., a large amount of clot with a higher number of thrombectomy passes, prolonged procedure time, reduced collateral supply and limited delivery of recombinant tissue plasminogen activator to the intracranial occlusion in comparison to more distal LVOs [3,4,5,6]. Anatomic variations of the circle of Willis might also be a factor influencing collateral blood supply and thereby the clinical outcome [2,5]. Common variants of the anterior circulation include aplasia or hypoplasia of the A1 segment of the anterior cerebral arteries (ACA). A1 hypoplasia is found in 10% and aplasia in 1–2% of postmortem examinations [7]. However, the meaning of this specific anatomic variant remains unclear concerning the outcome in AIS.

In this study, we analyze the influence of an insufficient circle of Willis collateral blood supply via the anterior communicating artery (AcomA) due to contralateral aplasia or hypoplasia of the ACA’s A1 segment on the clinical outcome in patients with distal ICA occlusions treated by endovascular MT.

## 2. Methods

We conducted a retrospective study of AIS patients treated endovascularly at 12 tertiary care centers between January 2015 and February 2021. Inclusion criteria were evidence of acute occlusion of the distal ICA on baseline imaging with subsequent treatment with MT. As institutional protocols varied, no exclusion criteria with regard to baseline imaging characteristics or perfusion parameters existed. Baseline parameters, technical features, angiographic and clinical outcomes were recorded. There were no limitations on procedural characteristics, including the use of different thrombectomy techniques and intra-arterial thrombolysis, which were left to the attending neuroradiologist’s discretion. According to neurological guidelines, patients received intravenous thrombolysis (IVT) whenever possible. Endovascular treatment was performed with approved MT devices, using stent-retrievers or large-bore aspiration catheters or a combination of both. Reperfusion success of the MCA territory was locally graded by a neuroradiologist according to the Thrombolysis In Cerebral Infarction (TICI) score [8]. Furthermore, the reperfusion success of both ACA territories was graded into complete and incomplete. Complications included the occurrence of post-interventional subarachnoid hemorrhage (SAH) and symptomatic intracranial hemorrhage (sICH). Clinical efficacy outcome was the rate of functional independence measured by the modified Rankin Scale (mRS) and defined as 0–2 at discharge and after 90 days. All National Institutes of Health Stroke Scale (NIHSS) and mRS grades were assessed by a certified stroke neurologist in each center.

**Figure 1:**
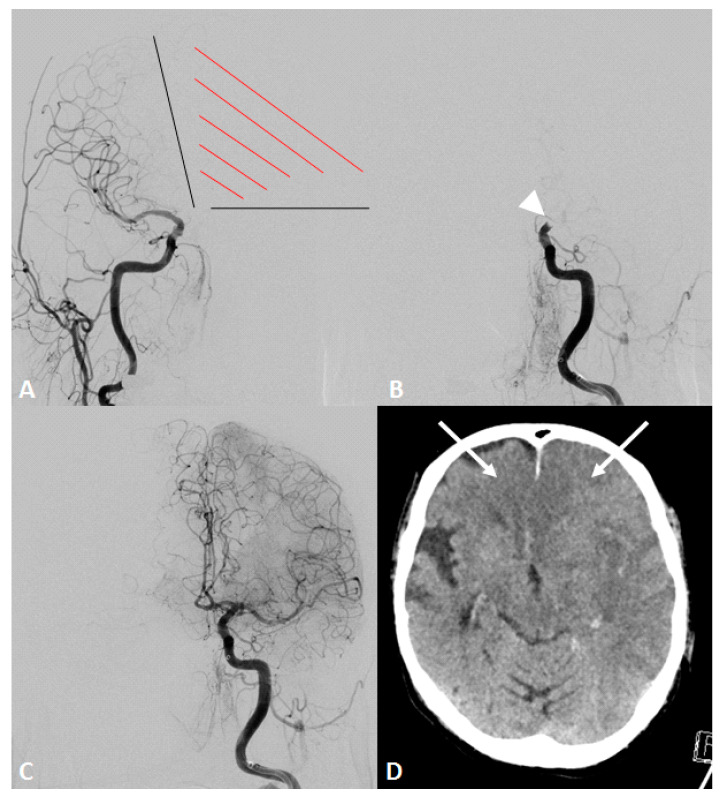
An octogenarian patient presented with an acute onset of right-sided hemiplegia and disturbed consciousness. Initial computed tomography showed an occlusion of the distal internal carotid artery (ICA) on the left side and no detectable infarction (not shown). Intravenous thrombolysis was initiated, and the patient was immediately transferred to the angio suite. (**A**) Initial angiogram of the contralateral ICA demonstrated an aplasia of the a1 segment and missing collaterals to the affected hemisphere, including both anterior cerebral artery (ACA) territories (red shaded area). (**B**) Angiogram of the left ICA showing distal ICA occlusion (white arrowhead). (**C**) Final angiogram after mechanical thrombectomy using two maneuvers with stent-retrieving showed a successful recanalization of the ICA with complete reperfusion of the middle cerebral artery (MCA) and both ACA territories. (**D**) Computed tomography on the next day demonstrated an expanded cerebral infarction in MCA (Alberta stroke programme early CT score of 0) and both ACA territories (white arrows). The patient died on the same day.

Evaluation of cerebral vasculature was obtained either from digital subtraction angiography (DSA) or from baseline imaging (including computed tomography angiography, CTA, or magnetic resonance imaging angiography, MRA). Primarily, the caliber of the contralateral A1 segment was evaluated on DSA if the vessel was depicted during the endovascular procedure, e.g., through contralateral ICA injection prior to MT to evaluate collateral supply (Figure 1) or on final angiogram through (retrograde) blood flow via AcomA. The absence of the A1 segment was defined as ‘aplasia’. If the vessel diameter of the contralateral A1 segment was reduced >50% in comparison to the ipsilateral site, it was graded as ‘hypoplasia’. Both terms together were defined as ‘functional aplasia’.

The extent of ischemic damage within the MCA territory was measured using the Alberta Stroke Program Early CT Score (ASPECTS) at admission and 24 h after MT. Additionally, an infarction within the ACA territory in both hemispheres was detected on post-interventional CT.

According to the guidelines of the local ethics committee, ethical approval was given for this anonymous retrospective study (ID 21-7237), which was conducted in accordance with the Declaration of Helsinki. A patient’s consent for treatment was obtained according to the individual, institutional guidelines. Due to the retrospective nature of the study, additional informed consent was deemed unnecessary.

### Statistical Analysis

Qualitative parameters are presented as numbers and percentages and compared using the Chi-Square or the Fisher’s Exact tests. Quantitative parameters are presented as a median with interquartile range (IQR) and compared using the Student’s *t*-test or the Mann–Whitney-U-test. Normality was evaluated with the Shapiro–Wilk test. Regression analyses were performed to adjust for confounding baseline parameters, reporting the adjusted odds ratios (OR) with 95% confidence intervals (CI). A logistic regression model was applied for binary outcome data and an ordinal regression model for ordinal outcome data. The overall goodness-of-fit was evaluated with the Cox and Snell measure of R^2^. Wald χ^2^ statistics were used to test the significance of the individual covariates in the respective regression model. All statistic tests were performed using SPSS software (IBM SPSS Statistics for Windows, Version 25.0, Armonk, NY, USA). A *p*-value <0.05 was considered statistically significant.

## 3. Results

One thousand and sixty-eight patients were treated with MT due to LVO of the distal ICA during the six-year study period. The median age was 77 years (range 67–84 years). Four hundred seventy-four patients (44%) were male. The median baseline NIHSS was 17 (IQR 13–21). The median groin puncture-to-reperfusion time was 58 min (IQR 34–97 min) with a successful (TICI ≥ 2b) and complete (TICI 3) reperfusion rate of 86% and 38%, respectively. Ninety days’ follow-up data were available for 778 patients (73%). Of those, 175 (22%) patients were functional independent (mRS ≤ 2), and the overall mortality was 45%.

Eighty-one (8%) individuals showed functional A1 aplasia contralateral to the distal ICA occlusion. Age, gender distribution and baseline ASPECTS did not differ between the patient groups with and without contralateral A1 aplasia (Table 1). The median time from symptom onset-to-groin and groin-to-reperfusion was shorter in patients with functional contralateral A1 aplasia with 175 (IQR 150–215) minutes (vs. 210, IQR 145–319 min, *p* < 0.005) and 48 (IQR 31–73) minutes (vs. 59, IQR 35–98 min, *p* < 0.05), respectively. The number of MT attempts and reperfusion success for both MCA and ACA territories did not differ. Patients with functional contralateral A1 aplasia were more severely affected on admission with a median NIHSS of 18 (IQR 15–23) than patients with a sufficient contralateral A1 segment (median NIHSS 17, IQR 13–21, *p* < 0.05; aOR: 0.672, 95% CI: 0.448–1.007, Wald = 3.706, *p* = 0.054). Post-interventional ischemic damage was larger in cases with functional contralateral A1 aplasia with a median ASPECTS of 5 (IQR 1–7) versus 6 (IQR 3–8) in the control group (*p* < 0.001, aOR: 1.817, 95% CI: 1.184–2.789, Wald = 7.476, *p* = 0.006). Furthermore, infarction within the ACA territory occurred more often in individuals with functional contralateral A1 aplasia with involvement of the ipsilateral territory (20/76, 26% vs. 110/961, 11%, *p* < 0.001; aOR: 2.482, 95% CI: 1.389–4.437, Wald = 415.078, *p* = 0.002), contralateral territory (3/76, 4% vs. 4/961, 1%, *p* < 0.05; aOR: 10.296, 95% CI: 1.941–54.608, Wald = 155.482, *p* = 0.006), and both ACA territories (8/76, 11% vs. 5/961, 1%, *p* < 0.001; aOR: 17.968, 95% CI: 4.979–64.847, Wald = 219.776, *p* ≤ 0.001). Functional contralateral A1 aplasia was associated with a worse clinical outcome in comparison to the control group. At discharge, 6/81 (8%) showed functional independence (mRS ≤ 2) vs. 194/965 (20%) in the control group (*p* < 0.05; aOR: 2.579, 95% CI: 1.086–6.122, Wald = 319.936, *p* = 0.032) and after 90 days, 5/55 (9%) individuals were independent vs. 170/723 (24%) in the control group (*p* < 0.05; aOR: 2.664, 95% CI: 1.031–6.883, Wald = 189.228, *p* = 0.043). In-hospital mortality tended to be higher with functional contralateral A1 aplasia (30/81, 37% vs. 254/965, 26%, *p* < 0.05; aOR: 1.506, 95% CI: 0.910–2.494, Wald = 222.324, *p* = 0.111).

Subgroup analysis showed that out of the 81 patients with functional contralateral A1 aplasia, both ACA territories were completely reperfused in 57 (70%) individuals. In the remainder, only an incomplete or futile ACA reperfusion was achieved. An incomplete or futile ACA reperfusion was associated with a higher in-hospital mortality (13/24, 54% vs. 17/57, 30%, *p* < 0.05; aOR: 2.778, 95% CI: 1.041–7.407, Wald = 4.156, *p* = 0.041) and tended to correlate with a lower median ASPECTS at 24 h compared to complete ACA reperfusion (2, IQR 1–7 vs. 5, IQR 3–8, *p* = 0.096; aOR: 0.464, 95% CI: 0.193–1.115, Wald = 2.948, *p* = 0.086). The clinical outcome at discharge and after 90 days did not differ between the groups.

## 4. Discussion

This multi-center study demonstrates that a functional A1 aplasia contralateral to a distal ICA occlusion is associated with a poorer clinical outcome after MT. Furthermore, our data suggest the importance of the complete reperfusion of both ACA territories apart from a successful recanalization of the ICA target vessel occlusion.

This is the first systematic report analyzing the relevance of this specific anatomic variant in AIS. Previously, case reports described the occurrence of bilateral cerebrovascular infarction within both ACA territories due to unilateral A1 aplasia [9,10]. Kang et al. previously showed this variant’s rarity in their study with 100 consecutive patients suffering from bilateral ACA infarction, but only one individual showed a combination of an ICA occlusion and an absence of the contralateral A1 [11].

It is obvious that an A1 aplasia of the uninvolved ICA-side is critical, as the protective collateral blood supply from the circle of Willis via AcomA is hindered. Thus, there is no antegrade filling of both ACA territories and subsequently no further collateral circulation through an opening of anastomoses between the ACAs and the occluded MCA [2]. Furthermore, a recent study from Pascalau et al. demonstrated that hypoplastic arteries in the Circle of Willis presented with a higher resistance to flow, which has a crucial effect on hemodynamic balance [12]. Taking this into consideration, it becomes clear that leptomeningeal anastomoses between both ACAs and ACA/MCA are more restricted in comparison to the normal anatomy of the circle of Willis. This might explain the main differences observed between the groups in our study: patients with functional contralateral A1 aplasia were affected more severely on admission, ischemic damage was the more significant and clinical outcome was poorer despite a lower time from groin puncture to reperfusion. The role of a diminished blood flow originating from the uninvolved side is reflected by the fact that aside from the lower ASPECTS (MCA territory), infarction of at least one ACA territory was more frequently observed. Furthermore, the reduced collateral supply might be unfavorable even for a subgroup of patients with a relatively favorable condition, who suffer from distal ICA occlusions but without the involvement of the ICA bifurcation (so-called “ICA-I” occlusions), as retrograde blood flow to the MCA territory via AcomA is severely impaired. However, in our study, a further differentiation into “carotid-I”, “carotid-L” and “carotid-T” occlusion was waived, as especially for patients with unilateral A1 aplasia, this anatomic condition could neither be clearly evaluated on DSA nor on cross-sectional imaging prior to the intervention as most of the institutes acquire only single-phase CTA in their routine stroke protocol. Although the distal ICA occlusion by itself is a heterogeneous pathology and can be classified more precisely [2], in our opinion, the exact extent of thrombus at the distal site plays a tangential role in this cohort as blood flow from the contralateral side is diminished due to A1 aplasia in any case of distal ICA occlusion pattern.

The higher in-house mortality expresses the importance of complete reperfusion in cases with incomplete or futile ACA reperfusion. Even if an influence on functional independence after 90 days could not clearly be demonstrated, the restoration of blood flow within the ACA territory should also be attempted during endovascular treatment as MT of distal ACA occlusions is meanwhile feasible and safe after the introduction of new device equipment, including small stent-retrievers and flexible reperfusion catheters [13].

Median time from symptom onset to the groin was shorter in patients with functional A1 aplasia. Although a distinct explanation cannot be given, it might be due to faster management, including direct patient transfer to a comprehensive stroke center and in-hospital transport as such patients are clinically severely affected. Optimally, the detection of a suspected insufficient ACA anatomy on baseline CTA in this cohort led to faster treatment decisions and accelerated patient transport to the angio suite.

The strengths of our study are the large number of consecutive patients included and the multi-center character. One major limitation is the retrospective design with a possible selection bias. It should be emphasized that institutional protocols varied between the centers with regard to baseline imaging, which might have influenced the decision for or against endovascular treatment. As a diminished collateral flow status might be responsible for the poorer outcome in patients with functional contralateral A1 aplasia, an assessment of a collateral score would be desirable. Collateral flow from the posterior circulation was not considered. Other anatomic variants such as accessory ACA, unpaired or azygous ACA as well as ipsilateral A1 aplasia were assigned to the control group, which might be negligible in this large cohort. Angiographic analysis of the reperfusion success was self-reported and may be less favorable after core laboratory adjudication.

## 5. Conclusions

A functional A1 aplasia contralateral to a distal ICA occlusion is associated with a poorer clinical outcome in acute stroke patients treated with MT. Furthermore, this study shows that this rare anatomic variant seems to play a substantial role in the development of large infarcts in MCA and both ACA territories. Fast and complete reperfusion of the affected territories should be attempted.

## Figures and Tables

**Table 1:** Characteristics between groups.

	Overall	Control Group	Functional Contralateral A1 Aplasia	*p* Value
**Baseline**				
Median age, years (IQR)	77 (67–84)	77 (67–84)	80 (69–85)	0.8
Male, *n* (%)	474/1068 (44)	438/987 (44)	36/81 (44)	1.0
Tandem occlusion, *n* (%)	172/1068 (16)	162/987 (16)	10/81 (12)	0.4
Large artery atherosclerosis, *n* (%)	329/1068 (31)	312/987 (32)	17/81 (21)	0.04
Cardio-embolic, *n* (%)	548/1068 (51)	502/987 (51)	46/81 (57)	0.4
IVT, *n* (%)	524/1068 (49)	475/987 (48)	49/81 (60)	0.04
Median baseline ASPECTS (IQR)	9 (7–10)	9 (7–10)	9 (7–10)	1.0
Median NIHSS admission (IQR)	17 (13–21)	17 (13–21)	18 (15–23)	0.04
**Procedural**				
Median time from onset to groin, min (IQR)	204 (145–307)	210 (145–319)	175 (150–215)	0.004
Median time from groin to reperfusion, min (IQR)	58 (34–97)	59 (35–98)	48 (31–73)	0.036
Median no. of MT attempts (IQR)	2 (1–4)	2 (1–4)	3 (2–4)	0.2
Successful reperfusion MCA (TICI ≥ 2b), *n* (%)	916/1068 (86)	843/987 (85)	72/81 (89)	0.4
Complete reperfusion MCA (TICI 3), *n* (%)	410/1068 (38)	376/987 (38)	34/81 (42)	0.5
Complete reperfusion ipsilateral ACA, *n* (%)	879/1001 (88)	816/920 (88)	63/81 (78)	0.3
Complications				
overall, *n* (%)	128/1068 (12)	115/987 (12)	13/81 (16)	0.2
SAH, *n* (%)	50/1068 (5)	45/987 (5)	5/81 (6)	0.5
sICH, *n* (%)	56/1068 (5)	51/987 (5)	5/81 (6)	0.7
**Outcome**				
Median 24 h ASPECTS (IQR)	6 (3–8)	6 (3–8)	5 (1–7)	0.001
ACA infarction				
ipsilateral	130/1037 (13)	110/961 (11)	20/76 (26)	<0.001
contralateral	7/1037 (1)	4/961 (1)	3/76 (4)	0.011
both	13/1037 (1)	5/961 (1)	8/76 (11)	<0.001
Median NIHSS discharge (IQR)	13 (5–42)	12 (4–42)	19 (10–42)	0.001
Discharge mRS ≤ 2, *n* (%)	200/1046 (19)	194/965 (20)	6/81 (8)	0.005
In-hospital mortality, *n* (%)	284/1046 (27)	254/965 (26)	30/81 (37)	0.037
90 days mRS ≤ 2, *n* (%)	175/778 (22)	170/723 (24)	5/55 (9)	0.014

## Abbreviations

ACA	anterior cerebral artery
AcomA	anterior communicating artery
AIS	acute ischemic stroke
ASPECTS	Alberta Stroke Program Early CT Score
CI	confidence interval
DSA	digital subtraction angiography
ICA	internal carotid artery
IQR	interquartile range
IVT	intravenous thrombolysis
LVO	large vessel occlusion
MCA	middle cerebral artery
MT	mechanical thrombectomy
MRA	magnet resonance imaging angiography
mRS	modified Rankin Scale
NIHSS	National Institutes of Health Stroke Scale
OR	odds ratio
SAH	subarachnoid hemorrhage
sICH	symptomatic intracranial hemorrhage
TICI	Thrombolysis in cerebral infarction
